# “Everyone says ‘safe sex’ but no one ever says ‘safe drugs’, you know?”: Perspectives on the intersection of drug checking services, drug policy and the overdose crisis

**DOI:** 10.1371/journal.pone.0321574

**Published:** 2025-04-17

**Authors:** Jeff Ondocsin, Lissa Moran, Daniel Ciccarone, Simon Outram, Dan Werb, Nicole Holm, Emily A. Arnold

**Affiliations:** 1 Center for AIDS Prevention Studies, Department of Medicine, University of California, San Francisco, California, United States of America; 2 Family & Community Medicine, Department of Medicine, University of California, San Francisco, California, United States of America; 3 Centre on Drug Policy Evaluation, St. Michael’s Hospital, Toronto, Ontario, Canada; 4 Division of Infectious Diseases & Global Public Health, UC San Diego School of Medicine, University of California, San Diego, California, United States of America; PLoS ONE, United States of America

## Abstract

**Background:**

Overdose deaths have continued to rise in the US despite heightened public attention and resources. Drug checking shows promise for integration into existing services for people who use drugs (PWUD) across North America. Amidst the backdrop of rising overdose deaths and emerging funds for harm reduction initiatives, this manuscript explores the landscape of drug checking services in North America and perspectives on improved integration with a diverse set of PWUD based in San Francisco and North American drug checking experts.

**Methods:**

Two separate samples of drug checking stakeholders, ‘providers’ and ‘clients’ were recruited. Providers participated in in-depth semi-structured qualitative interviews over Zoom on their experiences advocating for and operating drug checking services in the US and Canada. Clients were people who used drugs and lived in or commuted to the San Francisco Bay Area and participated in semi-structured interviews in November 2022. Interviews were transcribed fully and analyzed using thematic analysis methods.

**Results:**

Providers and clients identified ongoing instability in the North American drug supply that is exacerbating overdose risk while also identifying groups that would benefit from greater access to drug checking services. Both groups believed the paradoxical impacts of the fentanyl crisis at the core of drug checking services created barriers to the implementation and expansion of these services, hurting PWUD and their providers. Additionally, clients and providers reflected on the social and policy challenges to expansion and improvement of drug checking in their communities.

**Conclusion:**

Drug checking remains underleveraged, particularly with respect to the most vulnerable PWUD. Clients and providers contended that these services must become more responsive to an ever changing and dangerous drug supply in North America.

## Introduction

In the 2020s, ongoing rises in opioid-related overdose deaths have heightened awareness among health service professionals, community leaders, and politicians of the need for services to be provided to people who use drugs (PWUD) that will reduce the likelihood of overdose and other adverse drug-related outcomes. In the US, overdose deaths increased almost 15% from 2020 to 2021, rising to 107,622, of which more than 70,000 involved fentanyl [1], further extending the rises witnessed from 2019 to 2020 [2]. Deaths rose among all racial and ethnic groups [2], with relative increases observed in 2020 higher than any previous year since 1999 [3]. Mortality rate ratios from 2020 show overdose fatalities among older African Americans and American Indian/Alaska Native adults of all ages are even greater than Non-Hispanic White adults [4].Age-adjusted overdose rates from synthetic opioids other than methadone, which includes fentanyl and its analogs, continue to climb, as do overdose rates involving cocaine and psychostimulants [5]. These trends are mirrored by non-fatal overdoses, which rose more than 30% from January 2020 to January 2021, based on suspected drug overdoses in Emergency Departments [6]—likely a significant undercount of all non-fatal overdoses.

Fentanyl has been present in the US drug supply since at least 2013, becoming entrenched in the supply by initially adulterating and later supplanting heroin [7,8]. Polysubstance use, which can be both intentional and unintentional, is a significant driver of overdose [9,10] and awareness of this heightened risk has increased recently [10,11]. Specifically, fentanyl has co-adulterated stimulant-type drugs (e.g., methamphetamine & cocaine), and overdoses involving both fentanyl and stimulants have risen precipitously [12,13]. California (CA) has seen rising overdose deaths over this period. In 2018, one of the first years in which fentanyl-related deaths began to be observed in the state [14], CA had an overdose death rate of 12.8 per 100,000, slightly above the rates of the four years prior [15]. Each year since 2018 has seen notable increases, with 2021 being particularly deadly; 10,000 deaths and an overdose death rate of 26.6 per 100,000 [15]. Synthetic opioids accounted for nearly two thirds of deaths in 2021 [16]. San Francisco, like the rest of CA, saw the introduction of fentanyl in 2018 and overdoses began to rise, with 625 overdose deaths in 2021, 472 of which were attributed to fentanyl [17]. Since 2018, there have also been increases in cocaine- and methamphetamine-involved deaths, often in combination with opioids [17]. Deaths continue to rise, with 810 overdose deaths in 2023, higher than any previous year [18].

Drug checking services provide PWUD with voluntary information on the chemical composition of the drug compound in their possession. These services have been provided for several decades in Europe [19,20], but recently there has been a renewed focus on the role drug checking can play in public health surveillance and harm reduction services in North America. Particularly since the rise of fentanyl and other high-potency synthetic opioids in North American unregulated drug markets, drug checking has become a crucial technology for monitoring drugs at the point of use and improving risk communications to PWUD. Drug checking uses multiple modalities to test drugs with varying levels of sophistication. Each form of testing has related advantages and disadvantages with respect to analytical capabilities, cost of operation, time taken to test and provide feedback, and utility in respect to expectations [21–24]. Fieldwork for this study focused on two modes of drug checking, immunoassay strips and Fourier Transform Infrared (FTIR) spectrometry that were used in San Francisco, CA. Immunoassay strips, the earliest of which were fentanyl test strips (FTS), are the most widely used and recognized form of drug checking, and can rapidly indicate whether fentanyl (and a range of fentanyl analogs) is present in a sample with high sensitivity and specificity [25], typically offering detection in the ng/ml level [24]. FTS were initially designed for urine testing, and testing drugs put into solution directly is considered off-label use [24], potentially resulting in false positives at low cut-offs (below 40 ng/ml) or in the presence of other drugs [26]. Paradoxically, this is primarily how FTS are used currently. Additionally, FTS do not provide information on fentanyl concentrations or distinguish between analogs [24]. There has, however, been strong consensus that FTS are an accepted and useful modality among PWUD in many settings, and can promote harm reducing actions upon receipt of a positive test [27–29]. The success of FTS has led to further development of additional immunoassay strips as other drugs, notably benzodiazepines and the veterinary sedative xylazine, are increasingly found in the North American drug market [30,31].

Alternatively, spectrometry technologies can give a detailed breakdown of drug components, identifying emerging drug threats and adulterants, harmful byproducts of drug production and provide varying levels of quantification of the sample [21,22,24,32]. However, field-based drug checking has unique requirements in order to be effective, including remaining operable without excessive technical expertise, analyzing relatively small amounts of drugs and providing information in a time-sensitive manner [22]. One of the most commonly used approaches for field drug checking is Fourier Transform Infrared (FTIR) spectrometry, which produces spectra that can be analyzed and compared to spectral libraries to determine constituents contained within drug samples [32,33]. FTIR has some advantages for street- and mobile-based drug checking, particularly its accuracy in sample identification, small size and swift runtime (results provided within a few minutes) [24,33]. It is also preferred for its relatively easy operation, compared to more sophisticated spectrometry devices, and does not require specialized preparation of samples for analysis [33]. Particularly when optimized for street-based drug checking, FTIR is considered a qualitative drug checking modality, providing a breakdown of the main constituent ingredients. While it is possible to additionally provide semi-quantitative results, this is not believed to be particularly viable when not analyzed by expert technicians [32].

Although FTIR has notable strengths, it is not effective at detecting substances present in low concentrations (generally believed to be less than ~5% by weight) [33], a detection threshold that critically undermines the potential impact of FTIR, given the potential for overdose to occur with fentanyl samples under 5% by weight. It is also dependent on reference libraries, which require paid subscriptions and may not be particularly responsive to drug trends and detection of novel psychoactive substances [24,33]. Best practices in street-based drug checking services include the use of FTS alongside FTIR to offset the issue of low sensitivity [33]. In addition, there are significant disparities between states on the legality of drug checking technologies, and an overall lack of clarity about how existing laws may impact their use, complicating the rollout of these technologies [34].

Whilst the terminology and scope of drug checking services varies, the intended outcome frequently goes beyond solely providing chemical testing of drug samples. As such, Measham and Turnball argue “drug checking aims not simply to reduce drug-related deaths but also wider drug-related harm. Therefore its effectiveness should be measured not only by a reduction in drug-related deaths but by successful risk communications that enhance risk management and increase harm reduction practices.” [35] Amidst drastic increases in overdose-related mortality in CA, this study explores how currently deployed drug checking services in the state are being implemented to meet the needs of PWUD through qualitative interviews with a diverse set of PWUD based in San Francisco and drug checking experts in North America. In 2022, when this study was carried out, drug checking services in California remained in their nascent stage with few active providers. We deemed it worthwhile to supplement these few in-state provider interviews by drawing on the insights and experience of more established drug checking programs from locations across the US and Canada. Findings solely from the provider interviews have previously been published elsewhere [36]. This approach intends to understand how PWUD in the US think about and use available drug checking modalities and compare these findings with the experiences of drug checking experts working with these and other modalities across North America.

## Methods

Research consisted of recruiting and interviewing two different samples of drug checking stakeholders, referred to in this paper as ‘providers’ and ‘clients’. After initiating interviews with California-based providers, additional key informants were recruited through a convenience sample of the PI’s known contacts. Using theoretical sampling methods, these participants were asked to identify other potential participants. Providers were recruited between June 21 and November 17 2022, with two authors (DC & LM) conducting n=11 in-depth semi-structured qualitative interviews over Zoom with providers, who were clinicians, technical experts in drug checking, and researchers on their experiences opening and operating drug checking services in the US and Canada. Inclusion criteria for providers consisted of being 18 years of age or older, employees or volunteers at drug checking service sites, and being willing and able to provide informed consent. Eight providers worked in the US, two worked in Canada, and one worked in both countries. Two were clinicians, four researchers, and five were harm reduction providers, with some currently and others formerly involved in drug checking projects. Some purposive sampling was used among this group to include perspectives on overdose prevention through drug checking services. Interviews lasted between 45–60 minutes and were recorded and transcribed in their entirety. Informants were offered a $100 honorarium in the form of a gift card at the end of the interview. Informed consent was sought by interviewers and consent was provided verbally. Participants have been pseudonymized for anonymity.

The second set of key informants interviewed were the ‘clients’, who were people who used drugs and lived in or commuted to the San Francisco Bay Area. Two authors (JO and NH) carried out n=13 semi-structured interviews over a week in November 2022. Clients had to be at least 18 years old and currently using fentanyl, heroin or illicit stimulants through any means of administration. Exclusion criteria covered any individuals who were intoxicated or could not otherwise provide verbal consent to participate. This was a non-random purposive sample. Individuals attending harm reduction programs at four locations in San Francisco were approached, asked about their current drug use patterns and subsequently if they would be interested in participating in the study before being formally verbally consented. Some participants were recruited directly by harm reduction site staff informed of the recruitment criteria while investigators carried out other interviews. The San Francisco-based research team was familiar with these locations from prior fieldwork, had good relationships with organizational staff and benefited from access to private rooms for interviewing. Additionally, the presence of a relatively new drug checking service in the city provided a unique opportunity to understand whether clients were aware of this service and ascertain their thoughts about its approach. Several participants (n=3) were recruited directly from the drug checking site while accessing these services. Interviews lasted approximately 30–60 minutes and were audio recorded and transcribed in their entirety. Informed consent was sought and participants provided consent verbally to interviewers. Clients were provided a nominal cash payment of $25 for their time and expertise.

Material covered in the provider interview guide included experience opening and operating drug checking services, navigating logistical and political barriers for these services, scaling up programming, knowledge about drug checking technologies and experiences integrating drug checking program into existing services and settings. For the client interview guide, questions explored drug use trajectory, current use practices, experience accessing harm reduction services, and awareness and experience of using drug checking services in the city, focusing particularly on the use of FTIR and FTS. Clients were also read a description of spectrometry drug checking occurring in San Francisco, and asked questions specifically about this service, what barriers and facilitators to its use they saw and how the service might be combined with other services and agencies. Both providers and clients were asked whether they thought drug checking could reduce opioid-related overdoses. The study was conducted in accordance with the Declaration of Helsinki and approved by the Institutional Review Board of the University of California, San Francisco (IRB numbers 22–36262 on 22 April 2022 and 22–36640 on 12 September 2022).

Following publication of a paper focused solely on providers [36], analysis of the client material followed. Interviews were transcribed and checked for accuracy before being uploaded to the Dedoose qualitative software package. SO created an initial coding structure using the client interview guide as its basis; this was refined in subsequent discussion with the other authors. SO and JO coded all the client interviews separately and JO checked both sets of coded interviews for consistency. It is estimated that consistency between the two reviewers was approximately 85–90% (consistency was high as the coding scheme was based largely on interview questions). Four authors (EAA, LM, SO and JO) read both datasets, identifying key themes across both datasets that would provide a structure for a fuller analysis. Using the tenets of thematic analysis, authors reduced and organized the provider data into analytic memos exploring patterns, contrasts and disagreements across this set of interviews [37,38]. The team met weekly during this process, discussing the data in-depth to further refine these thematic areas. Key thematic areas identified from the provider interviews including topics such as embedding drug checking programs into existing harm reduction services and information deficits in different drug checking technologies were also integrated into the coding for the client interviews. Coded material was read and discussed by all authors while outlining this paper, and alongside the provider analytic memos, forms the basis of these findings.

## Results

We present our findings within the following five thematic areas:

(1) Complex dangers of fentanyl, polysubstance use, and adaptations to fentanyl in the drug supply;
(2) Social and policy contexts
(3) Assessing risk profiles;
(4) Fentanyl test strips and the challenge of focusing solely on fentanyl;
(5) Consumer knowledge and community overdose prevention;

### Complex dangers: fentanyl, polysubstance use, and polypharmacy contamination

Both clients and providers presented concerns about the US drug supply, emphasizing the dominant position of fentanyl in minds and marketplaces*—“we have fentanyl, which has dominated the opioids on the street. There is still some black tar heroin available, but it’s limited and harder to find....Opioid pressed pills are mostly fentanyl.” (Provider 1, US-based drug checking operator)—*while also recognizing a burgeoning scene of polysubstance use adjacent to and interacting with the fentanyl market: *“But I guess people are on all kinds of drugs, everything, you know? They’re on G, they’re on acid, they’re on speed, they’re on heroin, fentanyl.” [Client 1, polysubstance use, point-of-use drug checking experience]* Fentanyl overdose, both fatal and non-fatal, has rightly taken central priority in US policy debates, but these overdoses are also occurring within a context of intentional and unintentional polysubstance use not limited to fentanyl alone. Amidst greater polysubstance use and both intentional and unintentional polypharmacy contamination of the drug supply, there is a groundswell of health and social effects from both fentanyl use alone and polysubstance fentanyl use observed by both groups. For some PWUD in San Francisco, especially among those not using fentanyl, these effects were especially differentiated from periods when other opioids were dominant: *“…it seems like fentanyl is starting to dominate. It’s like you can see everyone on it, almost, it seems like. [chuckles] It’s like – I don’t know – a trip. […] It seems like they don’t…care, you know, anymore or something.” [Client 1]* Reports of unusual or unexpected effects when using drugs are common, often chalked up to contamination, allergic reactions, unwanted byproducts from production and occasionally to intentional poisoning. However, clients reported witnessing and experiencing unusual events that did not align with their lived, bodily, and social experience of opioid use: *“And when they do fentanyl, they act like they are fucking possessed. And I don’t know... Like weird sounds, strange movements. And then they have no recollection of it. And that doesn’t sound opiate-y to me. And it’s making my girl stand sideways. [chuckles] And I don’t like that. She doesn’t like that. I’d just like to know what’s in our drugs.” [Client 2, polysubstance use, point-of-use drug checking experience]*

This client also believed that a similar phenomenon was playing out in the stimulant market: *“A lot of people are going crazy, and they’re not doing more stimulants than they ever have. The stimulants aren’t good.” [Client 2]* Drug markets are known to fluctuate in terms of the quality and composition of the drugs being sold, with considerable idiosyncrasy at the street and local level, but perceptions from both groups indicated this instability has accelerated, complicating risk and health communications:

“…I think there’s a national recognition that what’s in the street drugs is not what we used to think it is and the way we’re seeing that play out on the ground. So, because of what we can now tell is in the street drug supply is changing the way that we interact with our medical colleagues, with our harm reduction colleagues, because what they’re facing is something unknown. It is something that we haven’t seen before. And that changes day to day.” (Provider 2, US-based researcher)

Both providers and clients were enthusiastic about drug checking’s potential role in both overdose prevention and improving our understanding of the drug supply outside of law enforcement channels. For clients, knowing what is in their drugs was on its face as basic as knowing the ingredients in food, and even more critical given the dangers of the marketplace:

“I just think it’s just too important to know what’s in your shit, to know what’s in your drugs. I mean we know what’s in our food, right? The packaging is all labeled and the ingredients are listed. It’s just too important, especially with drugs. Especially because we don’t know who’s making them. We don’t know exactly where they’re coming from. And every single one is different. Every week is different. Even if you buy it from the same person all the time, they’re always having something different.” [Client 3, polysubstance use, drug checking site experience]

The emergence of fentanyl as a desired product significantly complicates California and other states' policies that remain focused on fentanyl identification, particularly through the provision of FTS and, presumably, alongside the notion that people can choose to avoid fentanyl if it is undesired: *“So for people who are using fentanyl, all a fentanyl test strip can tell you is, like, yes, no, there is fentanyl or a fentanyl analog in this – in this drug. […] So for a person who uses fentanyl, they’re kind of, like, okay, well, I hope there’s fentanyl in this because that’s what I bought; you know?” (Provider 3, Canada-based drug checking operator)* This sentiment was borne out by the experience of clients who use fentanyl daily:

*Interviewer: “Do you have any concerns about fentanyl being in your meth?”*

*Participant: “I have concerns about no fentanyl being in my fentanyl.” [Client 2]*

Drug checking, while promising, remains in its nascent stages in most locations, and clients and providers are left to grapple alone with an uncontrolled drug supply, with many interventions, including various test strips, intended for point-of-use applications. Many PWUD, including nine clients in this study, have occasionally prioritized point-of-use methods to prevent overdose and adapted how they use fentanyl [39]. At the same time, because these adaptations are point-of-use changes, they may not always accommodate the nuances of an unstable drug supply: *“…the supply just keeps getting more potent and, like, more contaminated, and people don’t know, and it’s very hard to respond. […] and that is, like, precisely, you know, what is at the root of what is killing people.” (Provider 3)*

### Social and policy contexts

These findings are situated within the social and policy contexts in which our informants navigate the drug market and support services. Clients and providers based in the SF Bay Area were keenly aware of the politically charged discussion around drug use, homelessness and harm reduction services in the city: *“…people who don’t do like crystal or fentanyl and stuff who wouldn’t understand what we are trying to do with this [drug checking] because I feel that they would try to shut it down… they’re afraid of what they don’t know.” [Client 4, methamphetamine use, drug checking site experience]*

Indeed, some clients reported experiencing violent backlash and threats from citizens protesting the open-air services that many PWUD access and where drug checking for street-based populations is likely to be most effective:

“…one night we had a bystander, just a local resident who just came onto the property and start taking photos with an iPhone or something, just videoing everybody here and was making a big stink about us here. So, I think the police had to come get involved and escort this man out of the area. So, there could be some, […] Hostility. Yeah, judgment from the community.” [Client 3]

“Every outreach has… there’s a lot of anger on the streets, and a lot of people get upset about things. Violence is a big thing.” [Client 5, methamphetamine use, drug checking site experience]

Others thought that there would need to be a targeted outreach in order to acclimate some PWUD to the idea that drug checking could be a trusted resource, particularly with a service that is offered directly or in partnership with the city of San Francisco:

“People [have a] general distrust and suspicion of official type of stuff, whether it be government or corporate or etc. Especially in the homeless community, there’s a very big suspicion of any and everything in some cases…” [Client 6, polysubstance use, drug checking site experience]

Among clients who had previously used spectrometry drug services in San Francisco, there was extensive enthusiasm about the project, its worth in the community and many reported extremely positive feedback about their personal experiences. There was still widespread skepticism among clients, however, that drug checking services as described to them would be designed for their benefit, particularly if the service was being offered by the city:

*Interviewer: “Do you think this service was designed with someone like you in mind?”*

*Participant: “No. Maybe. […] I don’t think the city of San Francisco had me in mind for too many of their decisions.” [Client 7, polysubstance user, point-of-use drug checking experience]*

Clients thought that drug checking could be particularly impactful when coupled with other life-saving interventions, such as overdose prevention sites, especially given the financial and logistical constraints of how people purchase and use drugs in the city:

“I think, if they put that machine somewhere where you’re allowed to get high and you could maybe stop there first on your way to – like if there’s a safe addiction site and happen to have that machine in the room or in the lobby. […] Most people I know can’t afford to get large quantities of drugs of any kind. So, they’re not so... Yeah. I mean you’re not going to know past that little bit what’s in [your drugs]…” [Client 2]

Providers agreed, while emphasizing that there are many other factors than the drug supply that have notable influences on overdose rates: *“In my mind, the problems that contribute to overdose are prohibition, law enforcement harassment, and everything that surrounds that that creates a shitty drug supply and then prevents people from investigating it.”* *(Provider 4, US-based drug checking operator)* This provider believed that drug checking amplified and confirmed existing skills that PWUD have for drug discernment and risk identification but improves outcomes in specific circumstances: *“I would say that it’s not like the direct health education piece of drug checking that’s going to change people’s behavior and reduce risk. People already have these skills and knowledge of techniques. It’s that they can identify what is risky about their specific batch in that moment.” (Provider 4)*

They also grappled with the perceived gap between the legality of drug checking and the still-illegal nature of drug possession in most US jurisdictions, which raises interesting contradictions in the rollout of this service:

“…we were very intentional in Chicago, we used CDC funds that came through our state to purchase drug checking. And then, we invited the CDC, and we invited the people from the state to come check it out. And to actually walk through it, go ahead and do it, we photo documented the whole thing. What this means is that we just had our public health departments, federal and state public health departments, holding illegal drugs. Because this is what you have to do if you’re going to fucking check drugs, is you need to hold drugs.” (Provider 5, US-based drug checking operator)

This divide between drug checking and drug possession was front of mind for many clients and, particularly among those who had previously used spectrometry drug checking, these political and social hurdles were well understood: *“Everyone says “safe sex” but no one ever says “safe drugs” you know? Especially when it comes to politics.” [Client 5]*

### Assessing risk profiles

Both groups identified the fentanyl preference binary—those who use fentanyl and those who do not are using the same tools for identification, with varying motivations—as a potential point of tension in California and US drug policy. Drug checking services are often oriented toward the street-based user, but our participants identified many additional groups who were thought to be particularly susceptible to fentanyl-related overdose and would experience an outsize benefit from expanded availability of drug checking.

#### People new to fentanyl, weekend warriors and people who use stimulants.

Both groups identified new users of fentanyl as particularly susceptible to overdose, and more in need of specific information about the drug they are using: *“Overdoses are – usually people are just getting to know fentanyl, they’re just playing with it and then they – that’s how it happens.” [Client 8, methamphetamine use, no drug checking experience]* Providers thought people who use drugs casually, i.e., ‘weekend warriors’ as well as the opioid-naïve might reasonably stand to benefit from drug checking. However, these individuals may not be aware of the availability of this service in their area, or may be less likely to seek out services designed for street-based populations. Despite prolonged advertising campaigns, many individuals remain unaware of the possibility of fentanyl contamination of the drug supply or share drugs or pipes with others without knowing they also contain fentanyl: *“Because I didn’t know what fentanyl was the first few times. It was in my bubble. I hit it. I was like, “What is this?” I thought it was dust or lint or something in there. And I didn’t know because I never heard of people smoking heroin and meth at the same time in a bubble.” [Client 9, methamphetamine use, drug checking site experience]* Among opioid-naïve individuals, drug checking could be particularly advantageous for overdose prevention: *“I think with people who are opioid naïve, be it young kids who are experimenting, weekend warriors, people who are using substances like stimulants on the weekends, they just don’t have any opioid tolerance, so any amount of fentanyl in their substance, and that could rapidly lead to an overdose be that nonfatal or fatal.” (Provider 6, US-based clinician and researcher)*

For people who use stimulants, the same was purported to be true: *“I think drug checking does have a lot of benefit for people using other substances – using cocaine, methamphetamine, ecstasy, I think those are the – the people that it could benefit the most.” (Provider 6)* Practically speaking, this was a significant concern among individuals not trying to use fentanyl, but unwittingly exposed to it in another drug. One participant who often purchased drugs in the South of Market neighborhood of San Francisco reported witnessing two overdoses from using presumed methamphetamine where drug checking services may have affected this outcome: *“When I was down here, I had actually two different people OD on fentanyl because they did too big of a shot of meth. And it had fentanyl in it and they ODed on the fentanyl and had to be Narcanned. Stopped breathing and everything. They bad, bad stopped breathing. Turned blue.” [Client 10, polysubstance use, point-of-use drug checking experience]*

Weekend warrior-types were thought particularly well suited to handle the drug-related requirements and time constraints of drug checking:

*“So, you know, there’s more of the privileged drug using individuals who buy their drugs in advance or [are] only using their drugs on weekends, [and] aren’t dependent on the drugs that they’re using, and they can build drug checking into their drug using behaviors, and they don’t mind waiting, like, a day or two for their results. […] folks who are using, like, MDMA, or cocaine, or ketamine, or, you know, LSD, that type of thing… giving up, like, 10 milligrams is nothing essentially for them.” (Provider 3)*

However, this same provider identified this as a barrier to street-based and other precarious populations: *“But for folks who are at highest risk of overdose and who are using opioids, 10 milligrams is significant. That’s about $2 of their drugs, which is significant, and it’s like a tenth of their dose, and many of them also can’t wait for their results; right? Like, they don’t have the privilege of being able to wait a day to get their results back.” (Provider 3)*

#### People who use fentanyl.

While initiatives intended to mitigate fentanyl overdose are the primary pieces of US and state-level drug policy, these directives also equate overdose prevention with fentanyl identification and avoidance. While many PWUD want to avoid fentanyl, this is complicated by those who use fentanyl daily to manage opioid dependence. According to clients, avoiding overdose cannot always be the top priority for those who require an increasingly potent dosage to stave off withdrawal: *“If one of them dies, everybody else wants to get whatever he got. It’s like heroin, but intense,” [Client 7]* highlighting the vast heterogeneity of opioid tolerances that coexist in the same drug communities due to rapid fentanyl proliferation. Clients, however, evinced a particularly nuanced understanding of the ways in which drug checking might be most useful in preventing overdose among PWUD and ways to improve the service:

“…spectrometry doesn’t tell us how much, the amount of fentanyl that’s in there. It doesn’t give us a ratio of fentanyl to other drugs. […] if they’re smoking and they’re mixing benzos on top of that, then that’s going to add to your likelihood of overdose.” [Client 3]

The issue of concentration was also raised by providers. One suggested that more knowledge about variability in fentanyl concentrations was of particular importance and would produce better outcomes, and ultimately, improve public health warnings aimed at specific communities:

“So, that level of volatility in the concentration is very useful from a public health, kind of population health, and population overdose prevention perspective. Because we’re seeing massive amounts of variability […] And communicating that median variability can save people’s lives beyond simply just telling someone that this is how much fentanyl is in this particular sample.” (Provider 7, Canada-based drug checking operator and researcher)

### Fentanyl test strips and the challenge of focusing solely on fentanyl

FTS remain an important technology to use alongside spectrometry, as one provider noted their particular strengths: *“…the pros are one, that they are scalable because they’re relatively affordable. […] And they have a very low detection threshold. They’re the most sensitive technology that we have, and they’re the most scalable.” (Provider 5)* Contrarily, the low-concentration detection issue for FTIR, the spectrometry device currently deployed in San Francisco drug checking, is problematic for fentanyl detection, missing doses which may be sufficient enough to cause an overdose among the opioid-naïve: *“I will say that I’m not in agreement with the dismissiveness of the fentanyl test strip. I think that they’re a very meaningful and useful tool. And they continue to be a backbone of drug checking, including when there is advanced technologies.” (Provider 5)*

Clients described situations in which they relied on FTS to help them make decisions about purchasing drugs on the street: *“Actually I just used them yesterday. Luckily, I didn’t buy the heroin I was going to, because it tested for fentanyl.” [Client 10]*

Provider 5, however, noted several weaknesses of the current reliance on FTS, particularly among structurally vulnerable populations: *“The cons are that they seem simple enough that user error abounds, and people are unaware that there’s so much user error. And so, I think that the risk for false positives is real, but more so it’s user error than actual, actual true false positives. […] it takes time, and you need a little bit of space, and you need more than just the fentanyl test strip to use it. And so, anybody who’s street based, or on the run, or on the go might not be able to just take a moment to set up their fentanyl test strip.” (Provider 5)* Others pointed to the difficulty of using them before each use and how drug checking works in practice among PWUD, with many providing samples only *after* previously using them:

“Even the idea of using a fentanyl test strip every time you use is so unrealistic. I mean, first of all, people would need thousands and thousands of them all the time, every day. […] And then, second of all, that’s not how people use drugs. People don’t use a fentanyl test strip every single time they’re going to get high. […] the idea that that is how drug checking should be striving to be, like available for every instance of drug use, I mean even in the way that the advanced drug checking technologies are being used, is like very often people are bringing samples of stuff after they’ve been used…” (Provider 1)

Providers also emphasized that FTS have a role to play in overdose prevention and maintaining personal autonomy, particularly while spectrometry remains expensive and has high technical demands:

“And the thing I like the most about the fentanyl test strips is that they put the tool in the hand of the user. And that is where we always get the most benefit, is when they person has control over the technology and when it’s used and how it’s used. […] But the way that we can give them to people, it’s like this is why we give people naloxone instead of... We don’t have people have to come to Needle Exchange to have their overdose reversed, right? We’re giving the technology to people so that they can manage their own existence.” (Provider 1)

Providers believed FTS used in combination with spectrometry can provide more information than either modality alone. Drug checking providers also reported using benzodiazepine test strips and xylazine test strips to ensure these drugs are not also present in a sample.

### Consumer knowledge and community overdose prevention

Providers and clients identified several areas where spectrometry drug checking may indirectly contribute to overdose prevention. These included improving knowledge sharing and community-wide knowledge of drug trends, as well as creating innovative programming that fits well into other services. Clients believed that publicly-available drug checking could contribute to an informal community for knowledge sharing among PWUD, increasing their ability to discern between substances as well as relay information about good and bad actors among the dealing community: *“I think it’s also a good camaraderie that people are just talking about using drugs and sharing information with each other about every aspect of the drug, where they’ve gotten it, who they’ve gotten it from, who’s been good, who’s been bad, what the market is like, you know, how people are treating each other…” [Client 3]*

Still others appreciated the potential for improved community drug monitoring, not just at the point-of-use, but creating a base of knowledge that could better inform trends in substance use and the drug supply at the community or neighborhood level:

*Interviewer 2: “What do you like about the idea?”*

*Participant: “Well, I liked a lot about it. One, that it was available in the first place. Two, that it was not just doing its own thing. It was part of a larger network that was keeping track of what drugs were popping up on the streets and what their makeup was. I really like that that’s happening.” [Client 6]*

Some stated that the community networks encompassing drug checking services should also integrate other services that PWUD need: *“And then, when possible, just having them on-site. So, we at our needle exchanges have contingency management treatment. So, folks can get hooked up with that. We have medical care, wound care. We have Suboxone start. We have folks who can get you either same day or next day into the methadone clinic.” (Provider 4)*

## Discussion

The introduction of fentanyl and other contaminants to the North American drug supply are contributing to increased polysubstance use, whether intentional or unintentional, driving overdose mortality ever higher. This crisis has contributed to greater interest in developing a more nuanced understanding of the drug supply among both service providers and PWUD. Various drug checking modalities are being employed in North America to fill this need and provide feedback on drug constituents directly to users of these services. However, while fentanyl identification has been the *raison d’être* for the uptake of drug checking, providers and clients complicated this rationale by identifying several weaknesses that limit this approach, while simultaneously recognizing groups who would benefit from expanded drug checking. Spectrometry was thought to be particularly impactful and yet still underutilized as a means to understand both the drug supply and overdose patterns in a swift-moving drug market. Providers and clients were also acutely aware of the delicate social, legal and political context framing the debate around drug checking and how these programs and harm reduction services more generally are facing significant opposition locally and federally in North America.

Drug checking services as currently constituted are attempting to strike a difficult balance: provide detailed and relevant information on the drug supply that is both timely and actionable. But given the urgency of the North American overdose crisis, this may be the wrong lens for scrutinizing the success of drug checking services, particularly when they are already so underleveraged. Giving people more information about the drugs they are using is extraordinarily important and returns agency to individuals who are often marginalized—providers and clients were emphatic on this point. However, it also places all the responsibility for a tainted drug supply back on the individual using drugs in an illicit, unregulated market where their means of control and self-protection are limited. Indeed, research among structurally vulnerable populations in Canada has found strong sentiment against using drug checking technologies, due to factors such as having to give up drugs for testing, time constraints, ambivalence towards overdose and limited recourse in the event of an unwanted result [40]. While other research has shown people with the means to do so will dispose of drugs they find out are contaminated [35], or change how they use them [41], structurally vulnerable populations including street-based individuals and people with opioid use disorder may not be as able to take these actions. A better drug checking service means providing clients with more reliable information, including communicating knowledge about concentrations, contaminants and other harmful components of a drug sample. But it also means moving beyond the fentanyl binary as expressed by the participants in this study.

To that end, spectrometry and other drug checking programs that employ quantitative (e.g., drug concentration) as well as qualitative reporting (i.e., the psychoactive compounds and other adulterants present in a sample) can provide individuals with a strong basis for informed decision-making regarding use of unregulated or unknown drugs. Indeed, providers and clients in our study believed that there are significant populations of PWUDs who are at risk of overdose for whom knowing the components and concentrations in a drug sample would be meaningful, whereas simply knowing if fentanyl is present in a sample would not be. Recent research in Chicago supports our findings, where among people who use opioids, most expected fentanyl contamination and believed more specific information, including drug concentrations, specific drugs found in the sample and the presence of potentially harmful diluents, would be more useful [42]. Areas where fentanyl has not yet reached saturation, e.g., Europe, may markedly benefit from integrating drug checking programs into existing services if fentanyl begins to enter these markets.

While the technologies currently being deployed are possibly adequate for reducing overdose mortality, their individual weaknesses require multiple, overlapping methods to be used in combination to provide the most comprehensive and timely information to PWUD, clinicians and policymakers. Drug checking technicians should therefore use not one, but many kinds of testing strips (fentanyl, xylazine and benzodiazepine, etc.) to investigate the tainted supply and provide useful information. However, because test strips can only provide targeted qualitative testing of specific compounds and cannot indicate the range of analogs that might be present, they are inherently limited. Simply adding further test strips to a drug checking regime may not be terribly useful in the long-term as novel psychoactive substances enter the marketplace and contribute to an unstable drug supply. Current evidence also indicates they do not accurately identify the presence of carfentanil [25], a potent opioid that has periodically emerged in the drug supply to devastating effect [43]. As one provider noted, “There is just no perfect model or perfect technology at this point,” requiring providers and clients to make do with available tools. However, existing evidence [33] and the experience of providers in this study finds that using FTS alongside FTIR provides more information than either modality alone. Early indications from drug supply surveillance, such as crime lab data surveillance [44,45], have been promising and require greater study and implementation. The potential for aggregating drug checking data within and across communities about local trends and emerging substances of concern also requires further exploration.

There is an urgent need to devote financial and technical resources to providing drug checking services, much of which work is currently being advanced by harm reduction service providers themselves [46] without largescale federal or state support. California and other US states are beginning to see large amounts of money roll into state coffers from several opioid litigation settlements with pharmaceutical companies and drug distributors. The first of these, settled in 2021 with Janssen Pharmaceuticals and three drug distributors (McKesson, AmerisourceBergen and Cardinal Health) will see California receive $2.05 billion dollars through 2038 [47]. The settlement funds will be used for current and future ‘opioid remediation activities,’ defined as “care, treatment, and other programs and expenditures designed to (1) address the misuse and abuse of opioid products, (2) treat or mitigate opioid use or related disorders, or (3) mitigate other alleged effects of, including on those injured as a result of, the opioid epidemic including naloxone distribution, support and expansion of existing medication assisted treatments (MAT), research and myriad other uses” [48]. However, outside of a call to support ‘fentanyl checking’ and research into FTS and other novel methods, these funds are lacking a crucial support for drug checking services in the state and broader monitoring of the drug supply outside of law enforcement channels. However, a bill passed the California legislature and was signed by Governor Gavin Newsom to clarify the legality of drug checking services in the state and increase access to these services [49,50].

Potential limitations of this study include its focus on drug checking solely in the North American context and limiting the client portion to one week of data collection in San Francisco. Providers from across North America were included to understand how drug checking has been implemented in diverse settings, some of which data was not germane to this manuscript. Provider experience using FTS and FTIR was prioritized for this manuscript. Some participants were unaware of the availability of drug checking services and had not utilized these resources in the city. It would be useful for a follow-up study to determine how drug checking implementation has impacted PWUD in California now that these services have been available for several years. The recent introduction of these services meant most PWUD interviewed had not heard of or used them before, with the exception of point-of-use technologies. The inclusion of multiple sites in San Francisco for client recruitment, including an active site for street-based drug checking, was intended to capture perspectives of PWUD from across the city and the utility of drug checking to individuals using different drugs, including non-opioid drugs. Additionally, the familiarity of the study team with San Francisco-based harm reduction services and the substance use landscape helped reach thematic saturation with a relatively small sample size.

## Conclusions

Drug checking is a promising intervention for overdose prevention, albeit one that remains severely underleveraged, particularly with respect to the most vulnerable drug-using populations in North America. Providers and clients identified a need for these services to become far more mainstream in the US and Canada, democratizing access and pushing beyond the fentanyl binary, recognized as a limitation of the potential of these technologies and one that is challenged by the realities of substance use in North America. Law enforcement channels for detecting emerging drug threats and communicating these findings to the public have been inadequate in their response to the rapidly changing drug supply—leaving grassroots programs to confront the challenges of the unstable North American drug supply.
